# Multidrug-Resistant *Escherichia coli* Associated with Respiratory and Systemic Infection in a Domestic Rabbit in Romania: First Confirmed Case

**DOI:** 10.3390/antibiotics14090929

**Published:** 2025-09-14

**Authors:** Vlad Iorgoni, Livia Stanga, Ionica Iancu, Janos Degi, Ionela Popa, Alexandru Gligor, Gabriel Orghici, Bogdan Sicoe, Ioan Cristian Dreghiciu, David Purec, Paula Nistor, Bogdan Florea, Corina Kracunović, Viorel Herman

**Affiliations:** 1 Department of Infectious Diseases and Preventive Medicine, Faculty of Veterinary Medicine, University of Life Sciences “King Mihai I”, 300645 Timişoara, Romania; vlad.iorgoni@usvt.ro (V.I.); janosdegi@usvt.ro (J.D.); alexandru.gligor@usvt.ro (A.G.); david.purec.fmv@usvt.ro (D.P.); viorel.herman@fmvt.ro (V.H.); 2 Discipline of Microbiology, Faculty of Medicine, “Victor Babes” University of Medicine and Pharmacy Timisoara, Eftimie Murgu Square 2, 300041 Timisoara, Romania; 3 Department of Semiology, Faculty of Veterinary Medicine, University of Life Sciences “King Mihai I”, 300645 Timişoara, Romania; ionela.popa@usvt.ro; 4 Department of Veterinary Emergencies, Faculty of Veterinary Medicine, University of Life Sciences “King Mihai I”, 300645 Timisoara, Romania; gabriel.orghici@usvt.ro; 5 Department of Radiology and Imaging, Faculty of Veterinary Medicine, University of Life Sciences “King Mihai I”, 300645 Timisoara, Romania; bogdan.sicoe@usvt.ro; 6 Department of Parasitology, University of Life Sciences “King Mihai I”, 300645 Timisoara, Romania; cristian.dreghiciu@usvt.ro; 7 Department of Internal Medicine, University of Life Sciences “King Mihai I”, 300645 Timisoara, Romania

**Keywords:** *Escherichia coli*, rabbit, respiratory disease, multidrug resistance, septicemia, One Health, backyard farming

## Abstract

Background/Objectives: This report documents the first confirmed case in Romania of fatal pneumonia and septicemia in a domestic rabbit caused by multidrug-resistant *Escherichia coli*, highlighting both its pathogenic potential and One Health implications. Case Study: An 8-month-old male German Giant Spotted rabbit raised on a rural farm under poor husbandry conditions developed acute respiratory distress and died within 48 h. Post-mortem examination revealed severe pulmonary congestion, tracheal inflammation, serofibrinous pericarditis, and systemic vascular lesions. Bacteriological analysis confirmed *E. coli* from lung, trachea, and bone marrow samples. The isolate demonstrated strong Congo red binding, was confirmed by MALDI-TOF mass spectrometry, and showed resistance to beta-lactams, fluoroquinolones, tetracyclines, sulfonamides, macrolides, and phenicols, remaining susceptible only to aminoglycosides. PCR screening identified virulence genes (*fimH*, *papC*, *iutA*, *ompA*) linked to adhesion, immune evasion, and iron acquisition, with potential for horizontal gene transfer. Conclusions: This first documented case in Romania emphasizes the clinical threat posed by multidrug-resistant *E. coli* in rabbits and the importance of early diagnosis, improved biosecurity, and responsible antimicrobial use. The zoonotic and environmental risks in backyard farming underscore the urgent need for integrated surveillance. Alternative control strategies, including phage therapy and probiotics, should be explored to reduce reliance on conventional antibiotics.

## 1. Introduction

The global rise in multidrug-resistant (MDR) bacteria has become a critical public health issue, as emphasized by the World Health Organization. The growing number of infections caused by MDR strains, coupled with the decreasing effectiveness of available treatments, poses a serious threat not only to human health but also to animal populations affected by infectious diseases. Multidrug resistance is generally defined as resistance to at least three different classes of antibiotics [1,2,3,4,5].

Rabbit farming is practiced worldwide, both in industrial systems and small-scale backyard setups. However, this sector faces notable health challenges, particularly from respiratory diseases that can significantly affect animal welfare and productivity. Among the bacterial agents involved, *Escherichia coli* has emerged as an increasingly important pathogen in rabbits. Although commonly regarded as part of the normal gut microbiota, certain strains of *E. coli* have demonstrated pathogenic potential, being capable of causing systemic and respiratory illness [6,7,8,9,10,11].

The role of *E. coli* in rabbit enteric disease has often been overlooked, partly because these bacteria can proliferate opportunistically in animals suffering from diarrhea of any origin. Nonetheless, enteropathogenic strains, especially those expressing the eae gene, have been implicated in disease outbreaks. Several serotypes, such as O103, O103:H2, O15:H, O109:H2, O128, and O132, are considered clinically significant. Notably, healthy rabbits do not normally harbor these strains in their gastrointestinal tract [10,12,13,14].

Two clinical forms of colibacillosis are generally observed in rabbits, depending on age. In very young kits (1–2 weeks old), infection may cause acute, yellowish diarrhea with high mortality, often affecting entire litters. In weaned rabbits (4–6 weeks old), the disease presents similarly to enterotoxemia, with fluid-filled intestines and petechial hemorrhages on the serosal surface. Affected animals may die within 5–14 days or survive in poor condition. Diagnosis typically involves bacterial isolation on blood agar, followed by biotyping or serotyping of the strains, and sometimes electron microscopy to detect mucosal adherence. Severe cases usually do not respond to treatment; thus, culling and rigorous disinfection are recommended. Preventive strategies, such as feeding high-fiber diets, appear to reduce disease incidence in weanlings [15,16,17,18].

Beyond gastrointestinal illness, *E. coli* is also recognized as a major cause of respira-tory infections, septicemia, and systemic disease across various animal species. The bac-terium′s virulence is supported by multiple factors, including adhesins, invasins, sidero-phores, and toxins, all contributing to its capacity to colonize the respiratory tract and evade host defenses. Despite ongoing research, effective commercial vaccines are not yet available. As a result, antimicrobial therapy, commonly with enrofloxacin, trimethoprim-sulfamethoxazole, or doxycycline, remains the primary approach for disease management. However, the frequent and sometimes indiscriminate use of antibiotics has contributed to the rise in resistant strains and heightened public concern about the safety of rabbit-derived food products [19,20,21,22].

Environmental conditions play a critical role in the onset and transmission of respiratory infections in rabbit farms. Factors such as poor ventilation, high humidity, accumulation of ammonia, and inappropriate bedding or housing designs can predispose animals to respiratory distress and facilitate the proliferation of pathogens, including *E. coli* and *Pasteurella multocida*. Moreover, structural elements such as flooring and cage hygiene significantly influence the microbial burden in the respiratory environment [23,24,25].

Inadequate management practices, including overcrowding, nutritional deficiencies, and lack of biosecurity measures, are significant risk factors for disease outbreaks. Stress induced by improper handling or transportation also suppresses immune function and increases susceptibility to infections. In addition to *E. coli*, other respiratory pathogens such as *Pseudomonas aeruginosa*, *Staphylococcus aureus*, and *Bordetella bronchiseptica* have been isolated from rabbits. Furthermore, coinfections involving *Pasteurella multocida* and *E. coli* are commonly reported in severe cases. The presence of other pathogens, such as *Mycoplasma* spp., viral agents, and environmental stressors, complicates diagnosis and treatment efforts. The lack of comprehensive diagnostic protocols and laboratory access further hinders proper disease management [26,27,28].

The respiratory tract microbiota in rabbits is increasingly recognized for its role in immunity and pathogen resistance. However, the specific dynamics between commensal and pathogenic *E. coli* strains in the respiratory system remain poorly understood. The emergence of antimicrobial resistance and the potential for horizontal gene transfer between commensals and pathogens increase the complexity of treatment and prevention strategies [29,30,31,32,33,34].

This study presents the first documented case in Romania of a young domestic rabbit exhibiting severe clinical signs of respiratory infection, including dyspnea, nasal discharge, and lethargy, followed by septicemia and death. Post-mortem examination and microbiological analysis confirmed *Escherichia coli* as the primary pathogen, isolating it from the lung and bone marrow. The strain exhibited multidrug resistance to several classes of antibiotics, underlining the urgent need for improved monitoring and antibiotic stewardship in rabbit farming.

## 2. Case Study

This case study describes an 8-month-old male German Giant Spotted rabbit (Oryctolagus cuniculus) that was submitted post-mortem to the Faculty of Veterinary Medicine in Timișoara, Romania. The necropsy was conducted within the Infectious Diseases Department following the sudden and unexplained death of the animal on a rural farm located in western Romania. According to the owner, the rabbit had been reared in suboptimal husbandry conditions characterized by overcrowding, poor ventilation, and limited sanitary control. No prior clinical examination or veterinary intervention had been performed. In the 48 h preceding death, the animal displayed signs of lethargy, complete anorexia, progressive respiratory distress, and mucopurulent nasal secretions. The deterioration was rapid and culminated in unexpected death during the night before presentation.

**Figure 1:**
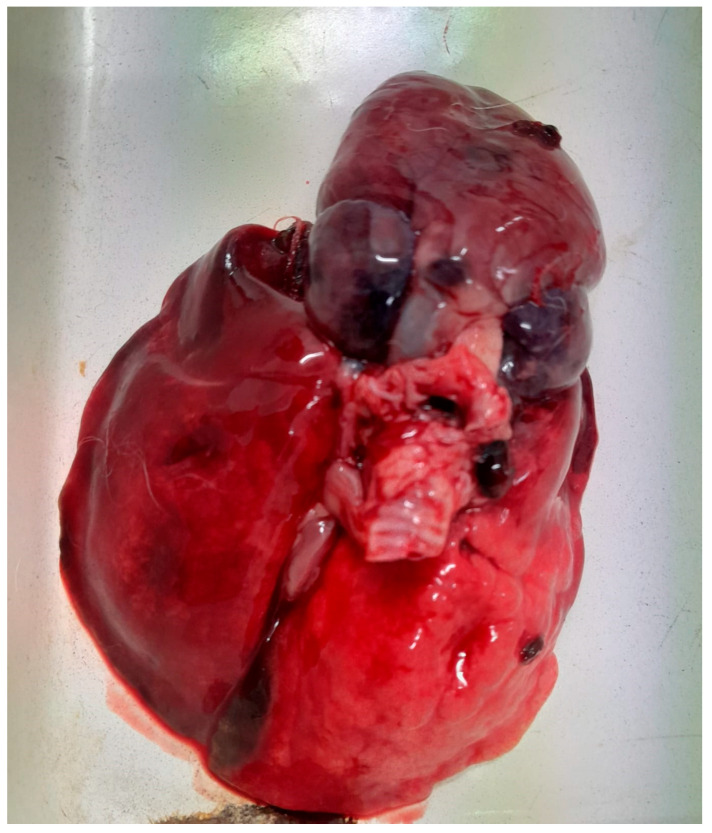
Lung presenting severe, diffuse congestion with multifocal dark red to black hemorrhages and moist, glistening surface indicative of edema, which consistent with acute fibrinous-purulent pneumonia likely linked to septicemia.

Gross pathological examination revealed marked alterations in multiple organ systems. The examined lung exhibited severe macroscopic lesions characterized by extensive consolidation, marked congestion, and focal areas of dark discoloration suggestive of hemorrhagic or infarcted regions. The surface appeared glossy and edematous, while the increased firmness of the parenchyma indicated an acute inflammatory process. Additionally, nodular formations and necrotic foci were observed, which was consistent with a potential progression toward suppuration or septic complications (Figure 1). These changes are compatible with a severe pneumonic process with possible systemic involvement. The tracheal mucosa was hyperemic and lined with thick, mucoid exudate that likely contributed to airway obstruction. In addition, moderate serofibrinous pericarditis was noted, along with generalized vascular congestion in the liver and spleen, suggesting systemic involvement. The bone marrow of the femur exhibited gelatinous transformation and congestion, findings compatible with hematogenous dissemination of infection. No digestive tract lesions, foreign bodies, or parasitic infestations were identified.

**Figure 2:**
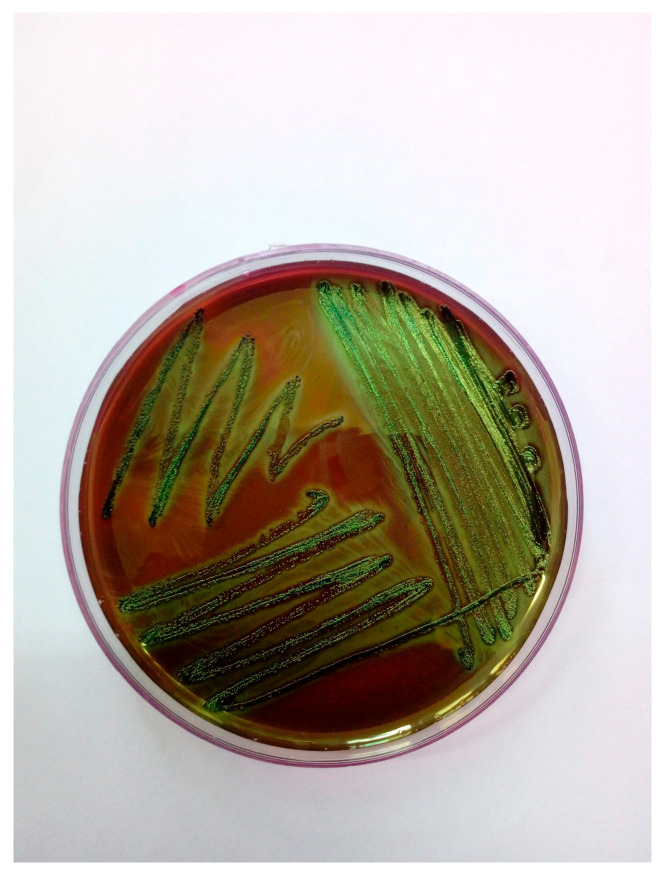
*Escherichia coli* colonies on EMB agar exhibiting a characteristic green metallic sheen.

**Figure 3:**
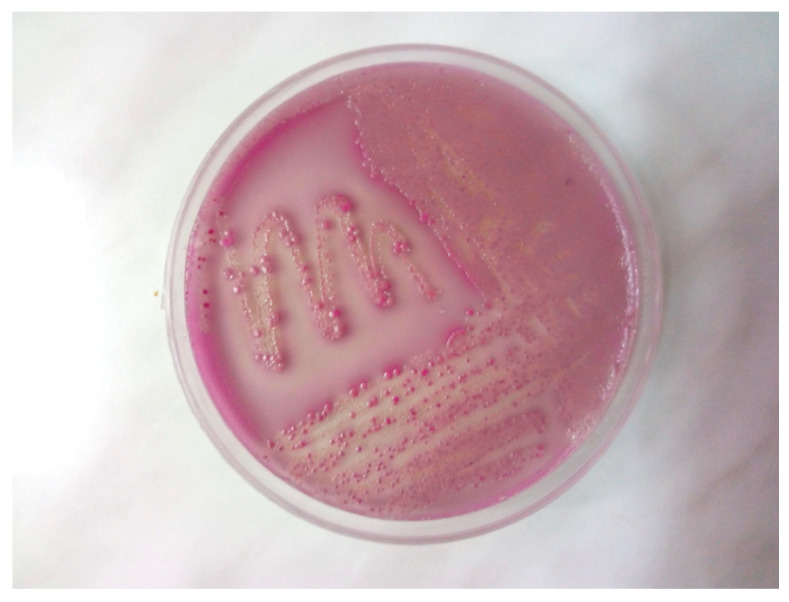
*Escherichia coli* colonies on MacConkey agar showing typical pink coloration due to lactose fermentation.

Samples collected aseptically from the lungs, trachea, and femoral bone marrow were cultured on Columbia agar with 5% sheep blood and MacConkey agar. Colonies were further subcultured on eosin methylene blue (EMB) and chromogenic media specific for *Escherichia coli*. After 24 h of aerobic incubation at 37 °C, pure growth of *E. coli* was observed in all samples, with visible colonies displaying a typical green metallic sheen on EMB agar and MacConkey agar (Figure 2 and Figure 3). In addition to conventional selective media, a chromogenic agar specific for *Escherichia coli* (CHROMagar Orientation, bioMérieux, France) was employed to facilitate rapid and accurate identification. This medium contains chromogenic substrates that target enzymatic activities characteristic of *E. coli*, primarily β-glucuronidase. Hydrolysis of these substrates released colored compounds, resulting in the formation of colonies with a distinctive mauve to purple coloration after 18–24 h of incubation at 37 °C. The differential color reaction allowed for reliable distinction of *E. coli* from other Enterobacterales, which produce colonies of different hues (e.g., blue, green, or colorless). In this case, the isolate developed the typical mauve coloration on chromogenic agar, corroborating the findings obtained on MacConkey.

**Figure 4:**
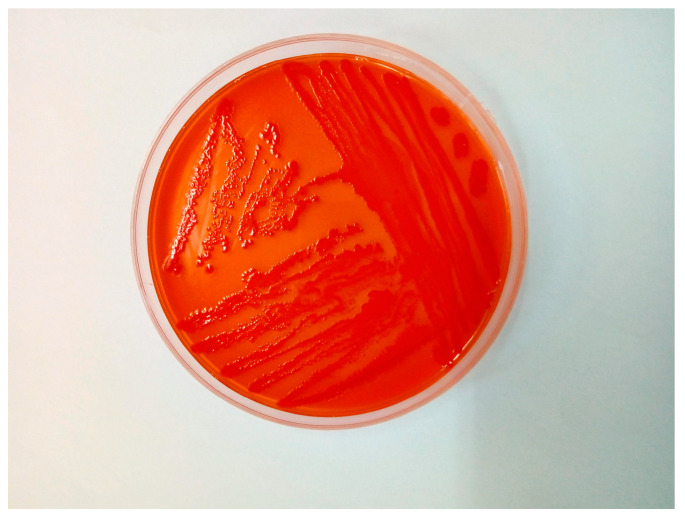
*Escherichia coli* colonies grown on Congo red agar demonstrating Congo red dye binding.

Congo red agar was used to assess virulence potential, and the isolate demonstrated Congo red dye binding, a phenotypic trait associated with increased pathogenicity (Figure 4). The identity of the strain was confirmed using MALDI-TOF mass spectrometry, yielding a high-confidence identification score of 2.18.

The isolation of *E. coli* from both pulmonary and bone marrow tissues confirmed the presence of septicemia. The bacterium was also recovered from the trachea, although this site was not considered sterile and its culture was not used as a definitive criterion for septicemia diagnosis. No other bacterial or fungal agents were identified, reinforcing the conclusion that *E. coli* was the sole etiological agent responsible for the fatal outcome. In order to assess the strain′s antimicrobial resistance profile, susceptibility testing was performed using the VITEK 2 automated system (bioMérieux, France) in strict adherence to the manufacturer’s protocols. Internal validation of the system was ensured using the control strain ATCC 25922, from Microbiologics Inc. (Saint Cloud, MN, USA), which demonstrated results within the accepted quality control ranges.

**Figure 5:**
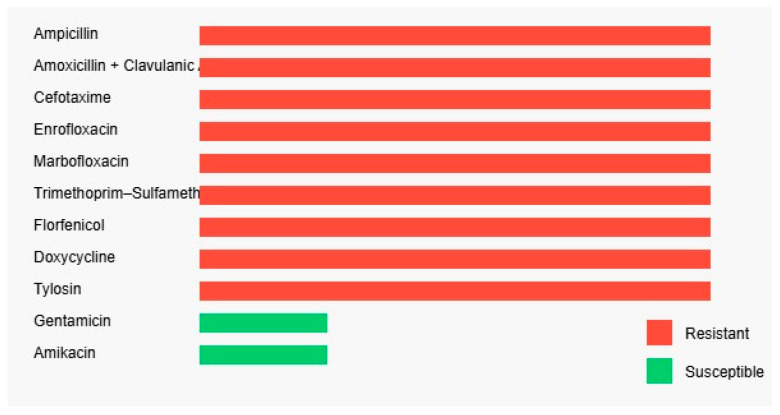
Antimicrobial susceptibility profile of the *Escherichia coli* strain as determined by the VITEK 2 system. The bar chart displays each antibiotic tested, with corresponding minimum inhibitory concentrations (MICs, µg/mL) labeled on the bars. Color coding indicates interpretation: red for resistant (R) and green for susceptible (S). The isolate exhibited resistance to a wide range of antimicrobial classes, including β-lactams (ampicillin, amoxicillin–clavulanic acid, cefotaxime), fluoroquinolones (enrofloxacin, marbofloxacin), sulfonamides (trimethoprim–sulfamethoxazole), phenicols (florfenicol), tetracyclines (doxycycline), and macrolides (tylosin). Susceptibility was observed only to aminoglycosides (gentamicin and amikacin).

The antibiogram revealed a multidrug-resistant phenotype. The isolate was resistant to beta-lactam antibiotics, including ampicillin (≥32 µg/mL), amoxicillin-clavulanic acid (≥16/8 µg/mL), and cefotaxime (≥64 µg/mL), as well as to fluoroquinolones such as mar-bofloxacin and enrofloxacin (≥8 µg/mL for both). High-level resistance was also observed for sulfonamides (trimethoprim-sulfamethoxazole ≥320 µg/mL), phenicols (florfenicol ≥32 µg/mL), tetracyclines (doxycycline ≥16 µg/mL), and macrolides (tylosin ≥16 µg/mL). The only retained efficacy was observed in the aminoglycoside class, where the isolate remained susceptible to gentamicin (≤1 µg/mL) and amikacin (≤4 µg/mL), indicating limited therapeutic options in a clinical context (Figure 5).

In addition to the phenotypic confirmation of pathogenicity, the genetic background of the *Escherichia coli* isolate was investigated to identify virulence determinants potentially contributing to its clinical impact. PCR-based screening revealed the presence of key virulence-associated genes, including fimH (type 1 fimbriae), papC (P fimbriae assembly), iutA (aerobactin receptor), and ompA (outer membrane protein), which are known to enhance bacterial adhesion, immune evasion, and tissue invasion. Molecular analysis of resistance determinants was conducted by PCR screening for major antimicrobial resistance genes. The isolate carried blaCTX-M and blaTEM genes, explaining its resistance to β-lactam antibiotics. Additionally, tetA and sul1 genes were identified, corresponding to the observed resistance to tetracyclines and sulfonamides. No plasmid-mediated quinolone resistance genes (*qnrA*, *qnrB*, *qnrS*) were detected. These findings indicate that the multidrug resistance phenotype of the strain is genetically supported by the presence of transferable resistance determinants.

The post-mortem findings, combined with the isolation of a multidrug-resistant *E. coli* strain from multiple sterile sites, confirmed the diagnosis of septicemia. No treatment was initiated in this case; however, the antimicrobial resistance profile strongly suggests that conventional empirical therapies would likely have failed. This case highlights the emerging threat of antimicrobial resistance in lagomorph pathogens and underscores the importance of early diagnostic efforts, responsible antimicrobial stewardship, and improved husbandry practices in rabbit farming. Furthermore, the systemic dissemination of *E. coli* and the degree of antimicrobial resistance observed may serve as a warning regarding the zoonotic and environmental implications of such strains circulating in backyard production settings.

Given the case-report nature of this study and the absence of a comparative or longitudinal dataset, no statistical analysis was performed. All microbiological findings were interpreted based on standardized diagnostic criteria. No metagenomic analysis was performed to rule out the presence of additional microorganisms. However, bacteriological culture consistently yielded pure growth of *Escherichia coli* from all tested samples, and no other bacterial or fungal pathogens were isolated. Furthermore, serotyping of the *E. coli* strain could not be conducted due to the lack of available reference O- and H-antisera. These aspects represent limitations of the present case study, as they restrict a more detailed epidemiological and molecular characterization of the isolate.

## 3. Discussion

This case illustrates the fatal outcome of an acute, systemic *Escherichia coli* infection in a rabbit raised under poor husbandry conditions. The presence of severe pulmonary lesions, tracheal inflammation, pericarditis, and bone marrow involvement confirms the systemic nature of the infection, while the isolation of *E. coli* from multiple sterile sites supports its etiological role. The lack of prior clinical intervention and rapid progression of clinical signs emphasize the importance of early detection and appropriate veterinary care in preventing severe complications.

Notably, the isolate exhibited a multidrug-resistant profile, showing resistance to multiple antibiotic classes commonly used in veterinary practice. This resistance pattern limits therapeutic options and raises concerns regarding empirical treatment efficacy, especially in rural or backyard settings where diagnostics are rarely performed. The findings reinforce the need for responsible antimicrobial use and improved biosecurity measures to minimize the spread of resistant strains and their potential zoonotic impact.

Suboptimal environmental conditions, such as poor hygiene, overcrowding, and inadequate ventilation, appear to play a key role in the onset and progression of the disease. Stress-related gastrointestinal hypomotility may favor the overgrowth of pathogenic strains and contribute to systemic infections [35,36].

The identification of enteropathogenic *Escherichia coli* (EPEC) O145:H2 in rabbits exhibiting acute diarrhea highlights the role of this pathotype as a significant cause of enteric disease in laboratory rabbit colonies. A strong association between EPEC-positive cultures and clinical signs supports its pathogenic role. The isolates harbored the eae gene and demonstrated a localized adherence-like pattern on HEp-2 cells similar to that observed in human infant EPEC infections, indicating shared virulence mechanisms across species. Therapeutic intervention with enrofloxacin proved effective, and its prophylactic administration prior to shipment significantly reduced morbidity and mortality. These findings underscore the zoonotic potential of EPEC strains in animals and reinforce the importance of molecular screening for virulence determinants in *E. coli* isolates from diarrheic cases [12,16].

In a study from 2016 conducted in Tai’an, 55 *Escherichia coli* strains were isolated from diarrheic rabbits on three farms and analyzed for antimicrobial resistance and genetic diversity. While all isolates were sensitive to ceftazidime, ceftriaxone, imipenem, and amikacin, high levels of resistance were observed to tetracycline (78.2%) and ampicillin (65.5%). Class I integrons were detected in 30.9% of isolates, and MLST analysis identified 13 sequence types, with ST302 and ST370 being the most prevalent. These results indicate a significant prevalence of antimicrobial resistance in *E. coli* from farmed rabbits, underscoring the need for responsible antibiotic use in rabbit production systems [37].

In the absence of effective vaccines, antibiotic therapy remains the main control strategy, although its efficacy is increasingly challenged by antimicrobial resistance. Alternative approaches such as bacteriophage therapy and probiotics offer potential benefits in reducing infection severity [38,39,40,41].

Prevention should focus on rigorous biosecurity measures, proper ventilation, balanced nutrition, and breeder education. Routine monitoring of resistance patterns is essential to guide rational antimicrobial use and reduce the spread of resistant strains in rabbit populations [42,43].

The detection of an *Escherichia coli* strain carrying multiple virulence determinants raises serious concerns regarding both clinical management and epidemiological control. The simultaneous presence of genes such as *fimH*, *papC*, and *iutA*, which are associated with adhesion, colonization, and iron acquisition, suggests a strain highly adapted to persist within the host and capable of causing severe systemic infections. The localization of these genes on mobile genetic elements increases the likelihood of horizontal transfer to other bacteria within the animal microbiota or to human-associated strains. From a One Health perspective, this genetic configuration underscores the necessity for integrated monitoring programs that not only assess antimicrobial susceptibility profiles but also investigate the molecular mechanisms underlying virulence. Such an approach would allow early detection of emerging high-risk clones and inform both veterinary and public health interventions. The virulence-associated genes identified in the *Escherichia coli* isolate from this case, *fimH*, *papC*, *iutA*, and *ompA*, have been previously reported in extraintestinal pathogenic *E. coli* (ExPEC) recovered from various animal species, including lagomorphs, companion animals, and poultry, as well as from human clinical cases. These genes are consistently associated with critical pathogenic traits such as adhesion to host epithelial surfaces (*fimH*, *papC*), iron acquisition under host-imposed nutritional limitation (*iutA*), and immune evasion (*ompA*) [44]. Their recurrent detection across diverse host species and geographical regions suggests that they represent a conserved virulence repertoire contributing to the capacity of *E. coli* strains to establish systemic infections. The presence of these determinants in isolates from rabbits has also been documented in previous studies, reinforcing the hypothesis that similar pathogenic mechanisms may operate across host taxa and highlighting the zoonotic potential of such strains within a One Health framework. Extraintestinal *E. coli* strains with multiple antibiotic resistances represent a major cause of infections in both humans and birds, posing significant public health and economic concerns [45,46].

## 4. Conclusions

The present case confirms the critical threat posed by multidrug-resistant *Escherichia coli* strains in domestic rabbits, particularly those raised in unsanitary and stressful environments. The systemic infection, confirmed by isolation of *E. coli* from both pulmonary and bone marrow tissues, resulted in a rapid and fatal outcome. The observed antimicrobial resistance profile indicates a significant limitation of available therapeutic options, raising concerns about empirical treatment failure. This underscores the need for improved diagnostics, biosecurity, and antimicrobial stewardship. Moreover, the potential zoonotic implications of such resistant strains in backyard settings necessitate integration of veterinary and public health surveillance under the One Health framework. Enhanced education for rabbit breeders, regular health monitoring, and exploration of alternative therapies such as phage treatment and probiotics are recommended for long-term disease control and public safety.

## Data Availability

The original contributions presented in this study are included in the article. Further inquiries can be directed to the corresponding author.
